# Economic geography II: The economic geographies of the COVID-19 pandemic

**DOI:** 10.1177/03091325231156926

**Published:** 2023-03-03

**Authors:** Andrew Leyshon

**Affiliations:** School of Geography, 6123 University of Nottingham, Nottingham, UK

**Keywords:** Covid-19, pandemic, crisis, inequality, uneven development, platforms

## Abstract

This is the second of three reports on economic geography. It focuses on research that addresses issues deemed to be both urgent and generative of crisis. This report focuses on the crisis created by the emergence of COVID-19. While the virus may have been novel, many of its implications were not, as several important processes of uneven development and inequality accelerated. The paper first determines the extent to which the emergence of a pandemic virus constituted a ‘crisis’, before examining some of the most salient economic geographical impacts of the COVID-19 pandemic. These include the rise of friction within the global economy resulting in significant disruption to global supply chains; the acceleration of the digital platform as an ever more dominant form of economic organisation; and how the pandemic deepened social inequalities and uneven development. The report concludes with observations about the emergence of what has been described as a post-pandemic polycrisis.

## I Introduction

This is the second of three reports on economic geography, focusing on research addressing issues deemed to be urgent and generative of a crisis. The first report (Leyshon, 2021) took as its subject the legacies of uneven development, with a particular focus on the contemporary United Kingdom where marked socioeconomic variations between places re-emerged as a political issue and – in the early 2020s at least – as a high-profile policy concern. This report is broader in scope and focuses on the crisis created by the emergence of COVID-19. Its level of transmission and severity meant that within three months the World Health Organization had classified it as a global pandemic. Many countries went into lockdown, with restrictions placed on movement and social contacts, producing significant disruptions to economy and society, with wide-ranging cultural and political implications, including concerns about civil liberties given the level of state intervention into everyday life (Kitchin, 2020). While the virus may have been novel, many of its implications were not, nor were many of the mitigations put in place to slow its spread. The social distancing measures introduced from 2020 resembled the techniques used 100 years earlier during the influenza pandemic of 1918–1920 (Spinney, 2017).

Given that the past 100 years has seen several pandemics, there are grounds to step back and interrogate the widely repeated claim that this pandemic, and the disruptions to economy and society that it caused, were ‘unprecedented’ (Davies et al., 2022). This report draws on recent research in economic geography and cognate fields to suggest that there was something distinctive about this pandemic that goes well beyond ‘recency bias’. Research has drawn attention to a wide range of consequences and responses to a rapidly developing and undoubtedly significant crisis and draws attention to the ways in which the spread of COVID-19 had significant implications for social equity, deepening existing lines of disadvantage at one turn, while bolstering privilege at another. Progress reports can necessarily only be contingent and provisional audits of relevant research, but this will be even more so: the object of this research is not only novel, but still evolving rapidly. At the time of writing, three years on from the start of the pandemic, there have been over 660 million cases of infection recorded globally, a cumulative total of 6.7 million deaths, while over 13 billion vaccine doses have been administered).^1^ The pandemic continues to progress although, due to higher levels of population immunity – a result of both extensive vaccination programmes and growing rates of prior infection – and the legacy effects of behavioural changes, most countries are treating COVID-19 as an endemic disease. In an endemic state, diseases reach a steady state of transmission, with cases no longer increasing exponentially. Infection still circulates within the population, with impacts on prevailing rates of morbidity and mortality, but – crucially – with a much-reduced risk of public health emergencies.

The novel nature of COVID-19, and the uneven global geography of vaccination and of public health capacity and response, means that the path from pandemic to endemic remains uncertain. Rates of transmission and numbers of new cases are likely to remain volatile. The perceived impacts of Covid-19 infection are seen to be less critical than at earlier stages of the pandemic, as measured by the number of hospital admissions with severe illness and deaths recorded with infection. Nevertheless, as high levels of uncertainty about the virus remain – which includes the possibility of further mutations that may be better able to escape the mitigating impact of the emergence of vaccines and antibodies (Wang et al., 2021) – the COVID-19 pandemic continues to present significant economic, social and political challenges, not least in adapting to the rapid transformations undertaken during the pandemic stage of the crisis and the shadow they cast. The damage that COVID-19 infection can inflict on previously healthy bodies is still being revealed.

The subject of this report is very much a crisis in process that continues to evolve and have significant impacts across a wide spectrum of activities and domains, with material consequences for economic geography. Later in the report, I set out some areas in which the emerging economic geographies of COVID-19 have been identified and explored, both within and beyond the sub-discipline. But first, I consider the extent to which the emergence of a pandemic virus constitutes a *crisis*.

## II COVID-19: What kind of crisis?

The first report drew on Janet Roitman’s work on the nature of crisis, in which she appraised the use and etymology of a term that was already widely used within political economy literature but had seemingly become ubiquitous in the wake of the Global Financial Crisis (GFC) (Roitman, 2014). The now taken-for-granted use of crisis as a noun in everyday commentary and analysis suggests that we’re living in an environment of multiple and overlapping crises. Roitman draws attention to the status of crisis as an historico-philosophical concept. Significantly, describing an event as a crisis is normative, in the sense that ‘it requires a comparative state for judgement: crisis compared to what?’ (2014, 4), while at the same time suggesting ‘a moral demand for a difference between the past and the future’ (2014, 8). It is this sense of change, of a rupture or sudden transition, which heralds a conjuncture with an uncertain or even negative outcome. It is this sense of an event being a pivot, located between the known past and an uncertain future, which prompts an occurrence to be identified as a crisis. In so doing, describing such an event as a crisis provides a narrative structure that navigates the past, present and future. In a more recent contribution, Roitman (2021) returns to her earlier work to assess the extent to which a pandemic qualifies as a crisis. As she states, at one level to even ask this question seems, frankly, to be absurd:

What else could a global pandemic be called, but a crisis? After all, human life is at stake … Nothing could seem more natural: the ultimate crisis for humans is not having an immune response to a virus. This is literally an existential crisis. However, we do have an immune response to Covid-19, which is how we developed a vaccine. So if the global pandemic is not an existential crisis that threatens the existence of the human species, what kind of crisis is it? (2021, 2 and 4).

A novel virus in contemporary society constitutes a crisis because it raises levels of morbidity and mortality which may overwhelm health services. Viruses such as influenza and the common cold (a coronavirus) circulate constantly in human populations and especially in the winter months in both the northern and southern hemispheres when it is colder and people spend more time indoors, where virus particles exhaled by infected carriers are less likely to dissipate, increasing the chances of infecting new hosts. Every year a seasonal increase in morbidity is accompanied by a rise in mortality, particularly among those with underlying health conditions and the elderly, who are more vulnerable due to comorbidities and depleted immune systems, so that additional strain is regularly put on public health services, including emergency services. And it is this feature, the extra load placed on public health services by a rapid increase in morbidity and higher levels of mortality from a novel and little understood virus, that explains why the advent of a pandemic signifies a crisis and demands state intervention. Not to intervene to modify behaviours and seek mitigations is to run the risk of a catastrophic collapse in all health care services which would damage public health even more, threatening the functioning of civil society.

It is as much the reaction to the perceived morbidity and mortality risks of a novel virus that created a crisis as a conjuncture in the way that Roitman describes. As Brinks and Ibert argue, identifying and naming a crisis is also part of a process by which state elites begin to organise and prepare themselves for action necessary to prevent a catastrophe, holding on as they do so to the possibility of an open and as yet undetermined future, but in so doing also create ‘a new reality in which uncertainty, urgency and threat predominate’ (2020, 284). It is this combination of an open future within the context of a threat that was not fully understood that helped frame the restrictions to everyday life enforced by states, as well as the behavioural responses of those individuals who sought to reduce their risks of contracting the virus. The disruption to normal behavioural patterns – both enforced and voluntary – served to unsettle and even halt some economic processes, while accelerating and deepening others. Moreover, the extent to which states and other authorities responded to the crisis through forms of lockdown and other mitigations varied (Brinks and Ibert, 2020; Buonsenso et al., 2021; Wang et al., 2020), which slowed the transmission of the virus by different degrees in different places, which circulated at varying velocities in relation to existing geographies of class, age and gender. Pandemics are always a crisis of geographical space because of the ways in which infectious diseases spread: they require bodies to come into contact with one another in physical space so that the virus particles can spread from host to host (Davies et al., 2022). But they are also spatialised crises because of the ways in which pandemic responses play out across economic, social and political geographies (Rose-Redwood et al., 2020).

The remainder of the report is given over to a discussion of three of the most salient economic geographical impacts of the COVID pandemic crisis

## III Friction

Pandemics scale through mobility. From an initial site of infection, most likely involving a virus crossing over from an animal host to a human through zoonotic spillover (Lakoff, 2021; Quammen, 2012; Wallace et al., 2015), a novel virus travels from host to host through a chain of transmission that relies on social interaction. Covid-19 emerged in a world that was far more integrated and interconnected than was the case in 1968 – the year of the ‘Hong Kong Flu’ pandemic – let alone the ‘Spanish Flu' pandemic of 1918. This greater level of integration was the product of increases in mobility over space due to advances in transportation. Once the virus escaped from its point of origin in Wuhan, China, it quickly circulated around the world. Time-space compression (Harvey, 1989) has been a key factor bringing about the globalization of production and consumption, entangling all manner of far-flung places through connections of money, people and materials (Sheppard 2016; Thrift, 2014). As Tooze notes, 2020 was the year in which we realised ‘that our ability to fly around the world vastly outpaced our understanding of what that interconnectedness entailed’ (2021, 65).

The growing integration of the global economy, enabled by advances in logistics and the seeking out of new production sites, especially in East Asia, has resulted in spatially extensive supply chains that have served to reduce costs in the long-term, but that in their extension have become inherently fragile and uncertain during periods of disruptions to ‘normal’ business conditions (Coe and Yeung, 2015; Cowen, 2014; Danyluk, 2018). Supply chains were disrupted throughout the pandemic because of the extraordinary measures enacted by governments across the world to close routes of capital circulation within their economies in a less than successful effort to prevent the virus being transmitted through those same routes (Davies et al., 2022; Tooze, 2021). Some places of production were closed to drive down infection rates while others had to change their ways of working to accommodate ‘social distancing’. Even where ‘essential’ places of work remained open, they were mostly forced to introduce costly and less productive ways of working. Other workplaces struggled to operate at all due to high staff absences because of illness, positive tests, or evidence of contact with a person testing positive. All of which served to cause significant disruption across various economic sectors. Aspects of this disruption were documented by several research projects ongoing during the pandemic.

The imposition of lockdowns in economies across the world which necessitated a closure of all non-essential places of business resulted in the full or partial closure of key parts of the global supply chains that produced, delivered and made available commonplace commodities, from factories, through transportation and transit, to shops and other retail units. There was a precipitous drop in both the supply and demand for all manner of consumer goods. Lawreniuk (2020), for example, revealed how the pandemic moved through Cambodia’s garment manufacturing sector, which was responsible for three-quarters of that country’s export earnings. Factories first suffered a shortage of raw materials as suppliers in China were closed as part of that country’s Zero Covid strategy, which sought to prevent any transmission of the virus. As physical retail outlets across the world were closed, Cambodia’s manufacturers then saw the markets for their products stall as global brands cancelled orders and used *force majeure* clauses to claim that contracts had been breached and refuse to pay for goods already made or entered into production. In response, Cambodia’s garment factories made up to a third of their employees redundant, making life even more precarious for a workforce more likely to be in debt to microfinance firms than the wider population (Lawreniuk, 2020). Similar problems of supply chain collapse were experienced in other garment manufacturing centres highly integrated with and dependent on global retail markets (Brydges and Hanlon, 2020). Even where production could be completed, fulfilling demand was constrained by staff shortages, due to illness and incapacity, and by increased biosecurity barriers at transport hubs and breakpoints. Delays in previously lean global logistical systems created long-lasting problems through the ‘bullwhip effect’; once supply and demand in established supply chain systems are disturbed, they take a long time to stabilise when various key components are out of place (Coe, 2021). To make things worse, the timing of restrictions varied between places as rates of infection ebbed and flowed, as did the appetites for infection risk expressed by different national governments, disrupting the flow of capital and commodities along chains of connection still further.

But in some areas, supply chain intensity increased, especially if linked to public procurement of personal protection equipment (PPE), the possession of which acquired a new priority across the world as governments sought to obtain the medical-grade masks, gloves, gowns and other equipment required by medical workers operating on the ‘front-line’ of the pandemic treating sick and ailing patients. While elsewhere workers were being laid off, in PPE supply chains employees were being taken on but, due to the urgency of demand, often with scant regard to issues of worker protection or rights. For example, Hughes et al. (2022) reveal how the explosion in demand for PPE led to an increase in incidents of forced labour in production sites in Malaysia, even when the buyers were governments – such as the UK – which had overt policies of using public procurement to directly combat modern slavery. The rise of forced labour was in part a response to a surge in demand for labour markets caused by the sudden unavailability of migrant labour, the flow of which was blocked as part of broader efforts to stem virus transmission.

Industries that were routinely dependent on migrant labour to regulate seasonal variations in demand, such as agriculture and tourism for example, were also impacted by the constraints in travel. Collins (2021) documented how, in places like New Zealand, which adopted a strict closed border policy to control the virus, industries dependent on migrant labour were severely impacted as were the migrant labourers themselves who were stranded in places where there was no longer any work due to the absence of international travellers. The closure of migrant labour markets also had the impact of disrupting the global flow of remittances which had serious implications for the recipients of such disbursements in many developing countries (Abel and Gietel-Basten, 2020).

It was urban economies, places of high population density and interactivity, that were perhaps the most badly impacted by the spread of COVID-19 (Nathan, 2021). While some essential services remained open, vast swathes of retail facilities were simply closed, while offices were largely emptied by work from home orders, with employees dependent on electronic means of communication to connect with others both inside and outside their organisations. The precipitous decline in work-day populations in urban areas had several impacts. First, as the occupancy rates of offices collapsed, so did their rental value, with implications for institutions and funds invested in commercial property as an asset class. The value of office property in New York was estimated to have declined by almost a third in 2020 as workers moved from the city to work from home (Gupta et al., 2022). Firms in urban centres everywhere looked to save money by re-evaluating their rental and lease agreements, many taking advantage of the collapse in use and demand to move to smaller accommodation, which was often both better-appointed and lower cost (Midlands Engine, 2022). Second, as working populations drifted away from urban centres, with signs that working from home facilitated by electronic communications would persist as a feature of the contemporary economy (Hobsbawm, 2022), there was a fear that one of the key dynamics animating leading agglomeration economies, face-to-face communication, would be impossible under COVID-19 mitigations undermining the competitiveness of locations that cultivate innovation and advantage through close and inter-personal interaction in place. For example, Zukin (2021: 5) voiced her fears for the long-term consequences of the pandemic for cities like New York that had invested considerable time and money cultivating a ‘tech ecosystem’ that ‘relied on face-to-face interaction – such as coworking spaces, hackathons and venture capitalists’ mentoring of start-up founders’ if the economy moved even partially online. A whole series of ancillary retail services that depended on office worker spending also collapsed, with signs that this income was being distributed to exurban and suburban centres where many office workers were now living and working (Gathergood and Guttman-Kenney, 2021). Concerns were similarly expressed about the future of financial centres that also relied on agglomeration economies and the importance of face-to-face interaction to communicate both information and emotion in fast moving markets. As the scale of the pandemic became clear, most banks fell into line with other employers and promptly sent their staff to work from home. But not all. Several banks in New York and London kept at least part of their trading floors open, which may have been at the cost of their employees’ health, but to the benefit of their companies’ financial bottom line (Buenza and Kent, 2020).

The exceptional profits were possible because during the early months of the pandemic all the indicators for the financial services sector were trending sharply downwards, with steep declines in the stock market values of firms right across the spectrum of services and activities (Wójcik and Ioannou, 2020). This collapse in confidence was informed by an expectation that slowing and even stopping economic activity would have serious consequences for global financial services firms that have been important drivers and conduits for the globalisation of economic activity over the past 40 years or so. However, the centrality of banking and finance to the contemporary economy meant that the threat of a catastrophic financial collapse caused governments to intervene through the extension of emergency monetary measures such as Quantitative Easing first undertaken in response to the GFC. These interventions supported and indeed strengthened the financial centres in which most of the world’s banking and trading activity took place.

## IV Platform ubiquity

The pandemic accelerated the rise of FinTech as the use of cash was discouraged for fear that the physical handling of notes and coins increased the risk of virus transmission (Wójcik, 2020). The use of FinTech apps for even trivial transactions helped to further embed platform practices into everyday life, just as platforms were increasing their sway over other areas of the economy. While the pandemic interfered with the movement of physical goods and created difficulties for organisations that relied on face-to-face activity, where ‘being there’ in physical space mattered, it also proved to be a boon to a different sector of firms already of such significance that some commentators had heralded the rise of a new kind of ‘platform economy’ (Grabher and van Tuijl, 2020; Kenney and Zysman, 2020; Langley and Leyshon, 2017; Peck and Phillips, 2021; Poell et al., 2019; Srnicek, 2016; van Dijck et al., 2019). Digital platforms, that provided intermediation in multi-sided markets, offered a solution to the problems of mobility and communication caused by pandemic mitigations. Videotelephony platforms, like Zoom and Teams for example, enabled office workers to relocate more or less *en masse* from their normal places of work to their homes while ensuring that their organisations remained functional. The shift to a widespread platform-intermediated, spatially distributed workplace was partially hindered by the supply chain problems referred to earlier, as the speed and effectiveness of the transition was impeded by a lack of appropriate equipment, with existing stocks of computers and laptops being quickly consumed, and the unsuitability of some homes as places of work. Long delays in supply chains developed, limiting the availability of key components, especially silicon chips. But after some adjustment, this new mode of platform-based work quickly became established (Reuschke and Felstead, 2020).

Meanwhile, broad spectrum online retail platforms, such as Amazon, and the gig economy platforms that focused on the delivery of food, helped households navigate the problems of lockdown and the enforced closures of shops and stores in the non-essential sector (Katta et al., 2020). Indeed, early in the crisis Amazon sought to position itself as a private sector version of the Red Cross, ‘keeping housebound citizens healthy, fed and watered – acting at times as if they were an extension of the emergency services’ (Lee and Nilsson, 2020). Platform models were incorporated directly in government attempts to manage the spread of Covid-19, with smart phone apps enrolled within what Dieter et al. (2021) have described as ‘pandemic platform governance’. As a result, while the pandemic saw the market capitalisation of most companies decline precipitously, the value of large digital platforms increased rapidly to become among the largest companies in the world. In the two years between the start of 2020 and the end of 2021, the market value of Apple increased by 123% to $2.9tn, Microsoft by 110% to $2.5tn, Alphabet (Google) by 108% to $1.9tn, Amazon by 85% to $1.7tn, and Meta (Facebook) by 60% to $936bn (Braithwaite, 2022). This rise in value was due to the ways in which the pandemic provided an accelerant to a form of capitalist organisation that was already colonising large swathes of economic and social life, so that platforms became critically important infrastructure for everyday life (Davies et al., 2022).

The growing importance of platforms as dominant modes of services delivery connects to a third implication of the pandemic, increases in geographies levels of inequality on a range of economic, social and health measures. Platforms may be multi-sided markets, but gaining access to them is not without cost, as equipment and bandwidth normally must be paid for. Moreover, as the pandemic hit, it became clear that there were significant differences between many of those who worked for platforms and those that benefited from them in other ways.

## V Pandemic inequality

Almost from the outset of the pandemic, research emerged anticipating that geographical inequality would deepen (Standring and Davies, 2020; Sokol and Pataccini, 2020). Initially there was an appeal to social solidarity, especially around health care, with calls to socialise the costs and risks of shutting down economy through measures such as furloughs and cash transfers, but over time the pandemic and the responses to it intensified ongoing transformations of inequality and inequity (Grove et al., 2022). The forms of economic development encouraged by neoliberal forms of government meant that some places and some communities were particularly vulnerable to the virus (Sparke and Williams, 2022). This could be seen in the way in which some places were, at various times during the pandemic, identified as COVID-19 infection ‘hot spots’, due to predominant forms of work and social conditions. Reporting on the high levels of Covid-19 infections in North East London in 2020 and 2021, Raval (2021) noted that

[h]igh levels of deprivation and job insecurity, vast income inequality, housing discrimination and medical disparities have long had a severe impact on the tangle of communities and ethnic minority populations that live in these boroughs. But when combined with the necessity to go to work, to take public transport and to share space in densely packed housing, they also provided the perfect breeding ground for a deadly virus.

Raval and others revealed the ways in which precarity of employment, manifest in low pay and little in the way of job protection, meant that workers who were unwell felt compelled to carry on working to generate income and maintain their job security. Similar relationships between poverty, precarity and the prevalence of COVID-19 was discovered in the global south. For example, Visagie and Turok (2020) revealed that despite the South African government introducing relief grants for the worst affected areas, the pandemic widened already existing economic and social divides between cities and rural areas on the one hand, and between suburbs and townships/informal settlements within cities on the other.

Suburbia benefitted as those workers who began to work from home and, as indicated above, took their spending power with them to the benefit of local vendors and the digital platforms that captured a growing share of retail consumption. Suburban workers were also generally better able to manage their exposure to the virus, so protecting their long-term health, while those that were homeowners also benefited from the rapid acceleration in the value of their properties during the pandemic, at least in the Global North (Adkins et al., 2021: 243). House price increases confounded predictions made in the immediate outbreak of the pandemic. However, strong government intervention, in part informed by emergency actions taken in the wake of the global financial crisis in 2008 (Tooze, 2021), not only kept the economy ticking over but saw an increase in the price of a wide range of assets, including houses. Thus, ‘quantitative easing, paid furlough schemes, enhanced unemployment payments and mortgage repayment holidays … not only prevented the liquidation of housing assets but also created the conditions for a fresh wave of asset price inflation’ (Adkins et al., 2021: 244). House prices increased fastest outside major urban centres, as those working from home engaged in what became known as a ‘race for space’, as families sought to move away from densely populated areas and/or to larger homes with outside space to better accommodate the pressures of working from home.

This process exacerbated the class-related outcomes of ill health. In addition to uneven class effects, these differential benefits were also intersectional. There were uneven intergenerational outcomes, as younger people were less likely to own houses, and more likely to be renting buildings of multiple occupation, so were often living in shared and smaller spaces, with more frequent interaction with relative strangers in domestic settings, increasing the risk of infection. Moreover, younger people were more disproportionately impacted by the closure of institutions as part of measures to control the spread of the virus. Some critics of school closure programmes, for example, argued that they were insufficiently evidence based and would wreak long-term generational damage on the economic prospects of children and young adults. This was further intensified by class differences. For example, Lewis et al. (2021) argued that, in the UK at least, repeated school closures brought about ‘an unprecedented intergenerational transfer of harm from elderly socioeconomically privileged people to disadvantaged children’ because children living in poorer areas were more dependent on the accumulation of educational capital to improve their life chances than those with access to other forms of capital. Given that only three countries refused to close schools during the pandemic – Nicaragua, Sweden and Taiwan – this was a pattern of structural disadvantage repeated on a global scale (Buonsenso et al., 2021). School closures also had a regressive gender impact, due to enduring differences in the burden of childcare. A study in the UK revealed that while women were more likely than men to have lost their jobs during the pandemic, this did not explain the unequal burden of additional childcare on women caused by COVID-19 school closure in the pandemic, as ‘the amount of additional childcare provided by women is less sensitive to their own employment than it is for men, leaving many women with (a lot) more child care’ (Sevilla and Smith, 2020: S170). Similar findings were found elsewhere, such as in the United States (Zamarro and Prados, 2021).

## VI Conclusions

While the immediate urgency of the COVID-19 crisis has declined since its outbreak at the end of 2019 and the breaking of successive waves of infection through 2020 and 2021, the slow transition from pandemic to endemic has not done anything to suggest that crisis is behind us. Rather, the shadow of the pandemic has seen an intensification of other problems, prompting a revival of the term polycrisis. First coined by Morin and Kern to refer to ‘interwoven and overlapping crises’ that combined to create a ‘complex intersolidarity of problems, antagonisms, crises, uncontrollable processes, and the general crisis of the planet’ (Morin and Kern, 1999: 74), it was revived by European Commission President Jean-Claude Juncker in 2016 to describe ‘the confluence of multiple, mutually reinforcing challenges facing the EU’ (Zeitlin et al., 2019: 973). More recently polycrisis has entered popular commentary ‘as a way of capturing a historical moment characterised by multiple global crises unfolding at the same time on an almost unprecedented scale’ (Derbyshire, 2023).

There are at least three dimensions to the emerging polycrisis, all of which reveal the influence of the pandemic. The first is economic, and which can be seen in the widespread growth of inflation as economies seek to recover and rebuild from the slowdowns and shutdowns of the pandemic. Several highly efficient and extensive lean supply chains have still not recovered, with shortages of key components evident. During the pandemic, the price of second-hand consumer goods increased, in some cases (like cars, for example), to match that of new goods, as a premium was placed on availability rather than condition. Costs have also been increased by labour shortages. Despite significant government interventions during the pandemic to keep people in paid employment through schemes such as furlough, or supported financially through cash transfers, millions of workers across the world lost their jobs through shutdowns. Reassembling these workers in place as economies have reopened has proved problematic. A shortage of migrant labour, the decision by many workers over the age 50 to leave the workforce – the ‘Great Resignation’ (Serenko, 2022) – and problems of ill health, including a proportion of the workforce suffering from the post-viral condition known as Long Covid, and others unable to work waiting for treatment in health services still coping with enhanced levels of morbidity caused by the pandemic, have combined to reduce the supply of available labour.

Inflation has been further accelerated by the second dimension of the polycrisis, which is geopolitical. Fuel prices began to increase in 2021 partly because of a long-term global shift away from coal to gas, including a steep rise in demand for liquefied natural gas as economies reopened after the pandemic (Sheppard, 2021). Prices increased sharply after Russia’s invasion of Ukraine in 2022 and its restriction of gas reserves for political leverage. The Ukraine war also disrupted agricultural production, given the importance of both Ukraine and Russia for food commodities such as wheat, and for the reliance upon this source of production in a set of emerging economies in Africa and Asia (Reed et al., 2022). At the same time, China’s strict enforcement of a policy of Zero Covid through to the end of 2022 continued to disrupt supply chains given the importance of the country as a global production site, further driving up prices. Its imposition of total lockdowns on areas with any infections was such that some companies were planning to reduce supply chain dependency on China, further fuelled by concerns about a possible conflict with Taiwan (Miller, 2022).

The third and final dimension of the polycrisis is environmental, which also takes us back to the origins of the pandemic. The likelihood of pandemics has been increasing (Marani et al., 2021), with Brenner and Ghosh (2022) arguing that pathways for new infectious disease have been created through the continued extension of ‘planetary urbanisation’ and the extension of neoliberal agribusiness into new parts of the world, bringing domesticated animals and agricultural workers into closer contact with ecologies within which novel viruses have long circulated, creating the opportunity for new zoonotic spillovers. This risk was identified many years earlier through the work undertaken under the banner of One Health, which sought to ‘address the complexities and interrelations that exist between human, animal and ecological health’ (Craddock and Hinchliffe, 2015: 1), advocating a multi-disciplinary research programme that incorporates a wide range of approaches that addresses ‘global and inter-species sharing of health concerns and interests’ (*ibid*) driven in large part by a recognition of the growing risk posed by zoonotic outbreaks and the need to combine social science and natural science approaches (Hinchliffe, 2015; Wallace et al., 2015; Woods et al., 2018).

Clearly, the environmental risk of the polycrisis goes far wider and is more extensive than the concerns of spillovers and the generation of new pandemics. Risks are inherent given indicators of climate breakdown that are increasingly apparent and that threaten to make the current organisation of economy and society unsustainable. The economic geographies of climate and environmental crisis will be the subject of my third and final report in this series.

## Note

1. WHO Coronavirus (COVID-19) dashboard: https://covid19.who.int/. Note that not all these deaths will necessarily have been caused directly by coronavirus infection leading to disputes about the ‘real’ death rate. For example, in the UK, Covid-related deaths are recorded as those that occur within 28-days of a positive Covid-19 test, which means that an unknown number of the deaths recorded by Covid-related would be more accurately described as ‘with Covid’ rather than ‘from Covid’. For a discussion see John Newton’s 2020 blog on the UK Health Security Agency website: https://ukhsa.blog.gov.uk/2020/08/12/behind-the-headlines-counting-covid-19-deaths/
